# Burden of diseases attributable to second-hand smoke exposure in Iran adolescents from 2009 to 2020

**DOI:** 10.1038/s41598-023-40058-z

**Published:** 2023-08-21

**Authors:** Hosna Janjani, Ramin Nabizadeh, Mansour Shamsipour, Homa Kashani, Mina Aghaei, Masud Yunesian

**Affiliations:** 1 Department of Environmental Health Engineering, School of Public Health, Tehran University of Medical Sciences, Tehran, Iran; 2 Department of Research Methodology and Data Analysis, Institute for Environmental Research, Tehran University of Medical Sciences, Tehran, Iran; 3 Center for Air Pollution Research (CAPR), Institute for Environmental Research (IER), Tehran University of Medical Sciences, Tehran, Iran

**Keywords:** Environmental sciences, Diseases, Risk factors

## Abstract

Exposure to second-hand smoke (SHS) is prevalent in many countries, but the problem’s scope is poorly understood globally, especially in developing countries. We aimed to estimate SHS exposure and its national and subnational burden of diseases in Iran, the second-largest country in the Middle East, during 2009–2020. The burden of diseases from SHS was estimated as disability-adjusted life years (DALYs) for adolescents (10–18) year’s non-smokers. Using comparative risk assessment methodologies, the calculations were based on disease-specific relative risk estimates with national and subnational SHS exposure data, and the uncertainty and sensitivity analysis was performed. The results of study showed that the trend of exposure to SHS is increasing in Iran. The highest DALY was related to lower respiratory infection (LRI), asthma, and otitis media, respectively. The national average asthma burden (DALY/100,000) has increased from 17.4 (11.8_23.9) in 2009 to 21.3 (13.9_30) in 2020, LRI decreased from 25.8 (21.5_30.2) to 19.8 (16.7_23.1), and national average burden of otitis media (DALY/100,000) has increased from 3.1(1.9_4.6) to 3.9(2.4_5.6). The increasing trend of otitis media and asthma DALYs attributable to SHS exposure in Iran requires more attention from policymakers to protect the population.

## Introduction

Second-hand smoke (SHS), or environmental tobacco smoke (ETS) is one of the most frequent indoor air pollutants worldwide^1–4^. According to the World Health Organization (WHO), almost eight million people die each year from tobacco-related diseases. Around 1.2 million people have died as a result of SHS exposure, with 1.091 million of them living in low and middle-income nations^5^.According to the Centers for Disease Control and Prevention (CDC), there is no safe level of SHS exposure; even brief exposure can affect human health. The International Agency for Research on Cancer (IARC) has listed second-hand tobacco smoke as a “Group 1” carcinogen (a known human carcinogen), and it has been shown to have many adverse health effects on adults and children^6^. Based on sufficient scientific literature, SHS is linked to ear infections, asthma, respiratory symptoms (such as coughing, sneezing, and shortness of breath), respiratory infections (bronchitis and pneumonia), and sudden infant death syndrome (SIDS). Furthermore, SHS can cause lung cancer, stroke, and heart disease in adult non-smokers^2,7^. Risk perception of SHS improved efforts to develop measures to prevent SHS exposure. Smoke-free policies prohibit smoking in indoor spaces, and designate public areas that have been broadly applied in workplaces, public venues, and transportation. Although smoke-free policies are becoming more common, more than 80% of the world's population remains unprotected^8^. So, it seems the world's large population is less likely to be protected by smoke-free laws, and people are at least at risk in public places and the workplace^9^. Xi et al. considered the SHS exposure in young adolescents in 68 low-income and middle-income countries and reported the overall prevalence of SHS exposure as 55.9% (2006–2013); the findings highlight the importance of improving tobacco prevention strategies and programs among young adolescents in low and middle-income countries^10^. In addition, Zhang et al.^11^ studied the prevalence of active and passive tobacco smoking among Beijing residents. They revealed that tobacco smoking prevalence is exceptionally high (44.74%) in Beijing, and nonsmokers suffer from SHS critically^1^. According to Carreras et al.^6^ in a systematic review study, the SHS exposure burden of diseases for several countries, especially the Middle Eastern and African countries, is not yet available. The highlighted literature review showed that SHS studies in exposure assessment and preventive measures in underdeveloped, middle, and low-income nations have been neglected. In Iran, smoke-free policies were approved following the World Health Organization Framework Convention on Tobacco Control (2003) to protect the population from the adverse health effects of SHS. Still, no special measures were taken, and studies showed the high prevalence of SHS in Iran’s population^12,13^. It seems care should be taken to reduce exposure to SHS in Iran and countries with similar status. Therefore, study of the exposure to SHS and its attributed burden is helpful for protection from SHS exposure and provides the most important objective and evidence for determining the demands of the health care system. Estimation of the burden of disease based on DALY is becoming more and more recognized as a crucial tool for supporting health-related decision-making. To our knowledge, no study has considered the SHS burden of diseases in Iran, the second-largest country in the Middle East, and epidemiological studies in this area are limited in developing countries. So, the present study was conducted to determine the exposure to SHS and estimate its attributed burden in young adolescents from 2009 to 2020. In addition, the details of the exposure estimation methodology in the present study can be useful in worldwide studies where information on the Middle East is sparse. This study provides enough details and evidence in this area for future actions to improve indoor air quality.

## Method

The present study quantified the second hand smoke exposure and various important health outcomes and was approved by the ethics committee (IR.TUMS.SPH.REC.1399.122) of Tehran University of Medical Sciences. In addition, all methods were performed in accordance with the relevant guidelines and regulations, considering the editorial and publishing policies of the scientific reports journal. In summary, this study can be presented in three phases to meet its main objectives. Phase I: exposure assessment, exposure to second-hand smoke was estimated by conducting a systematic review. Phase II: data Imputation, missing values of SHS exposure prevalence were predicted using statistical and modeling methods. Phase III: estimation of the burden of diseases attributable to SHS exposure, we used the comparative risk assessment method to estimate the burden of diseases attributable to SHS exposure^14^. The main assumptions in the present study were as follows: (1) the population attributable fraction, which is calculated using the percentage of people exposed to the relevant pollutant and the relative risk of disease associated with exposure, is used to determine the contribution of a risk factor to disease. It is defined as the proportional decrease in disease or death that would occur if exposure were reduced to zero. (2) the best estimate of RR from meta-analyses or systematic reviews. (3) a nonlinear regression function is considered for changes in exposure to second-hand smoke in the provinces.

### Exposure assessment

A systematic review was conducted to determine the SHS exposure of the Iranian population during 2009–2020 according to the Preferred Reporting Items for Systematic Reviews and Meta-analyses (PRISMA) guideline. The details of the systematic review were published in the previous study^12^. Findings of the systematic review regarding the exposure to SHS were not satisfying considering the age group of young adolescence, so national or provincial reports were considered. The principal investigators of nationwide health surveys were contacted for further information or access to data based on age, gender, and province subgroups. Following the above literature and national report searches, three national school-based nationwide health surveys were approached for access to data. These school-based nationwide health surveys were conducted in Iran as the national survey of school student high-risk behaviors (2009–2010, 2011–2012, and 2015) and were titled CASPIAN III, IV, and V studies. The data were gathered respectively for 5570, 9800, and 10,869 students aged 10–18 years in CASPIAN III, IV, and V. Students were selected by multistage random cluster sampling in urban and rural areas of 31 provinces in Iran. Eligible schools for CASPIAN surveys were stratified using the bank of Ministry of Education information, and they were selected randomly. In the included schools, students were selected randomly. Questionnaires were prepared in Farsi based on the WHO Global School Health Survey, and the students filled out the self-administered questionnaires at school under the supervision of health care professionals^15–17^. It is worth mentioning that written informed consent has been obtained from all subjects and/or their legal guardian(s) in these surveys.

### Data imputation

Data on socio-economic factors such as literacy, household expenditure and income, cost of tobacco products, smoking, urbanization, employment, and the development index were obtained from the National Statistics Institute and literature. The highlighted socio-economic factors were considered for creating new uncorrelated variables using principal component analysis (PCA). Eigenvalues > 1.0 were considered significant, and subsequently, varimax factors (VFs) and three main components were obtained for further analysis. A multiple regression analysis of these three main components with exposure to SHS was performed in provinces grouped based on changes in exposure to SHS smoke (Fig. S1). Then the missing data were imputed based on the multiple regression analysis equations (Tables S2–S5).

### Estimation of burden of diseases attributable to SHS exposure

#### Health effects selected for the estimate of burden of diseases

Exposure to SHS is associated with many adverse health outcomes^6,14^. In this study, we considered the three main health outcomes related to SHS exposure with strong evidence, including asthma (ICD10: J45–J46.9), lower respiratory infections (LRI) (ICD10: A48.1, A70, B97.4–B97.6, J09–J15.8, J16–J16.9, J20–J21.9, J91.0, P23.0–P23.4, U04–U04.9), and otitis media (ICD10: H70–H70.9). Also, the effect estimate for exposure to second-hand smoke were considered in terms of relative risk (RR) (Table 1)^6,14^.

**Table 1:** Health outcomes included in this study.

Outcomes	Description	Recommended risk (95% CI)
Asthma	Incidence of acute otitis media	1.32 (1.23–1.42)
Lower respiratory infectious (LRI)	Incidence of acute lower respiratory infections and admission to hospital	1.54 (1.40–1.69)
Otitis media	Incidence of otitis media	1.32 (1.20–1.45)

#### Population attributable fraction (PAF) calculation

Population attributable fractions (PAFs) were calculated based on exposure prevalence and relative risk.estimates, and the theoretical minimum risk exposure level (TMREL), using Eq. (1).

1 $${\text{PAF}} = \, \left[ {{\text{P}}\left( {{\text{RR}} - {1}} \right)} \right]/\left[ {{\text{P}}\left( {{\text{RR}} - {1}} \right) + {1}} \right]$$

where P is the proportion exposed to SHS in the specified population, RR is the relative risk for an outcome in the specified population.

#### Estimation of burden of diseases

In the present study, the population-attributable burden of diseases was estimated for adolescent non-smokers aged 10–18 years. So the smoking burden of diseases was subtracted from the overall burden of diseases, and the burden of each outcome was considered in the nonsmoker population^18^. Equation (2) was used to estimate the burden of three highlighted health outcomes in non-smokers.

2 $${\text{B}}_{{({\text{ns}})}} = \, \left( {{\text{B }} - \, \left( {{\text{B }} \times {\text{ PAF}}_{{{\text{sm}}}} } \right)} \right) \, \times \, \left( {{1} - {\text{p}}_{{{\text{sm}}}} } \right)$$

where B_ns_ is the total burden, in disability-adjusted life years (DALYs), of nonsmokers, p_sm_ is the active smoking rate, and PAF_sm_ is the population attributable fraction of active smoking. For each considered outcome, attributable burden of disease (AB) was calculated by multiplying the population attributable fraction (PAF_SHS_) by the overall burden due to that disease (B) following Eq.^14^:

3 $${\text{AB}} = {\text{PAF}}_{{{\text{SHS}}}} \times {\text{B}}_{{{\text{ns}}}}$$

The average population of three national censuses during the study period with an interval of 5 years was considered the population for the DALY calculation. Results were calculated at the national and subnational levels, and estimates of the overall burden of each outcome during the study period were obtained from the Institute for Health Metrics and Evaluation (IHME)^19^. Descriptive statistics were used to summarize the SHS exposure prevalence from 2009 to 2020. Since the DALY values for 2020 have not been estimated yet, we predicted these values using a linear regression model (the details are provided in Table S7). The burden of diseases attributed to SHS in the specific age group in Iran was reported as the 90% uncertainty intervals (5th and 95th percentiles). For each case, 500,000 iterations were estimated at national and sub-national levels during 2009–2020 using R statistical software (version 3.5.1) using ‘mc2d’package which provides a complete framework to build and study Two-Dimensional Monte-Carlo simulations, aka Second-Order Monte-Carlo simulations. ArcMap 10.3 used to create maps. Sensitivity analysis was performed using Oracle Crystal Ball version 11.1.2.4 to determine the variables that affected dispersion and uncertainty. DALY of asthma, otitis media, and LRI, RR for second-hand smoke, the prevalence of second-hand smoke, and RR for smoking, with an almost triangular distribution considered in the sensitivity analysis. The triangular distribution was assumed in order to be as conservative as possible.

### Ethics approval and consent to participate

Our project was approved by the Tehran University of Medical Sciences (IR. TUMS. SPH. REC. 1399. 122).

## Results

Based on the Table 2, it seems the trend of exposure to SHS is increasing in Iran (the details of the exposure trend are described in the previous study)^12^. Based on the increasing trend of exposure, the national average exposure to SHS in 2020 (48.47 ± 15.43%) is higher than in other years. The highest exposure to SHS was predicted at 66.02% for Sistan and Baluchestan provinces in 2020, and the lowest exposure was 15.28% in Ilam in 2009.

**Table 2:** Descriptive statistics of exposure to SHS in Iran adolescents 2009–2020.

Year	Mean	Std deviation	Variance	Minimum	Maximum	Range
2009	36.93	15.68	246.04	13.89	83.10	69.21
2010	39.16	9.82	96.59	24.20	58.07	33.87
2011	40.09	7.69	59.22	27.11	54.48	27.37
2012	44.85	9.45	89.45	20.13	83.33	63.21
2013	41.95	4.29	18.47	32.28	51.69	19.41
2014	42.88	3.88	15.11	32.28	53.74	21.46
2015	43.65	6.08	37.08	29.78	56.99	27.21
2016	44.74	6.51	42.39	32.28	57.83	25.55
2017	45.67	8.54	73.03	32.28	59.88	27.60
2018	46.60	10.72	115.01	29.32	61.93	32.61
2019	47.53	12.97	168.33	25.73	63.98	38.25
2020	48.46	15.26	232.99	22.13	66.02	43.89

**Figure 1:**
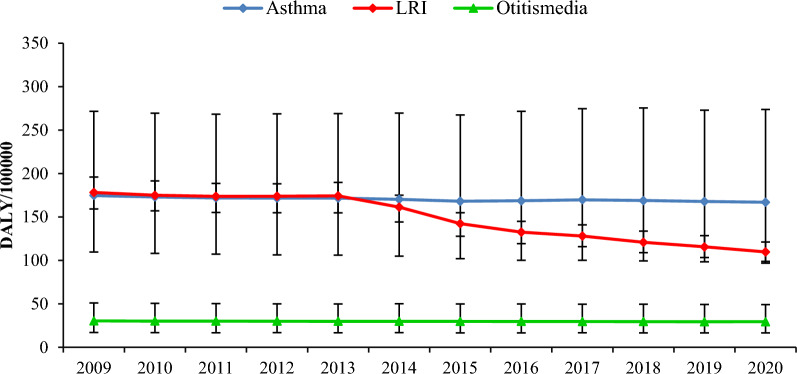
DALYs due to asthma, LRI and otitismedia in Iran adolescents 2009–2020.

Based on Fig. 1, the trend of asthma, otitis media, and LRI burden in Iran adolescents has been increasing during 2009–2020. In addition, according to Table S8, the highest rates of considered health outcomes burden were related to LRI, asthma, and otitis media, respectively.

**Figure 2:**
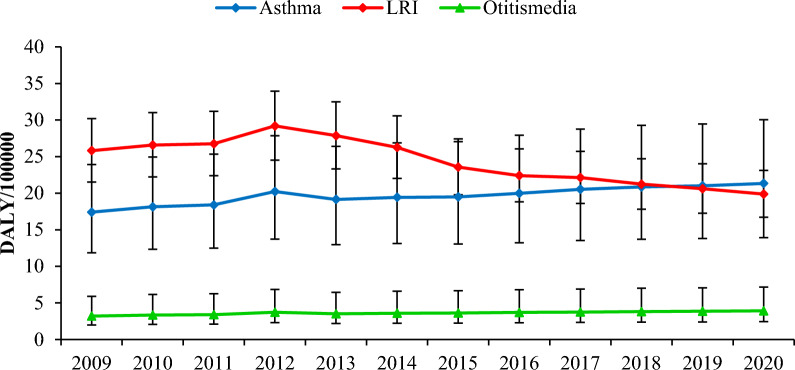
DALYs due to asthma, LRI and otitis media in relation to exposure to SHS in Iran adolescents 2009–2020.

According to the Fig. 2, the trend of asthma and otitis media DALYs in relation to exposure to SHS has been increasing and the trend of lower respiratory tract infection DALYs has been increasing (2009–2012) and then decreasing during 2012–2020 in Iran adolescents. The highest DALY was related to respiratory tract infections, asthma, and otitis media, respectively (Table S8).

**Figure 3:**
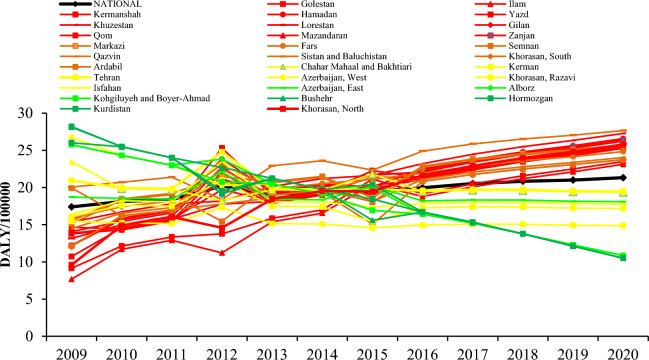
DALYs due to asthma in relation to exposure to SHS in Iran adolescents at the provincial level in 2009–2020.

According to Fig. 3, at the provincial level, the trend of asthma burden in 19 provinces of Iran is increasing (Ardabil, Fars, Gilan, Golestan, Hamadan, Ilam, Kermanshah, North Khorasan, South Khorasan, Khuzestan, Lorestan, Markazi, Mazandaran, Qazvin, Qom, Semnan, Sistan and Baluchestan, Yazd, Zanjan). In nine provinces, (Alborz, Chaharmahal Bakhtiari, West Azerbaijan, Bushehr, Hormozgan, Kohkiluyeh and Boyer Ahmad, Kurdistan, Kerman and Tehran) asthma burden decreased, and no changes were observed in three provinces of, East Azerbaijan, Isfahan and Khorasan Razavi. More details are provided in Table S9.

**Figure 4:**
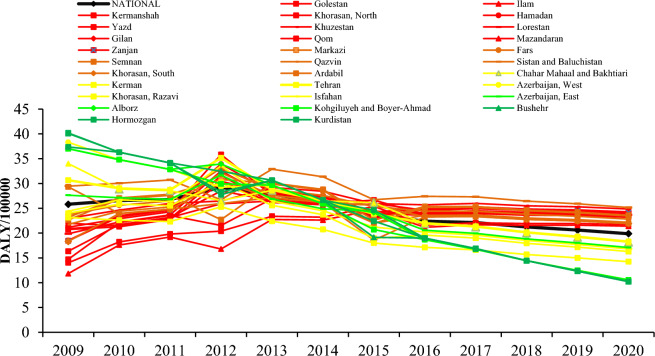
DALYs due to LRI in relation to exposure to SHS in Iran adolescents at the provincial level in 2009–2020.

According to Fig. 4, at the provincial level, the trend of LRI burden in 13 provinces of Iran is increasing (Gilan, Golestan, Hamadan, Ilam, Kermanshah, North Khorasan, South Khorasan, Khuzestan, Lorestan, Mazandaran, Qom, Yazd, and Zanjan). In 14 provinces (Alborz, Sistan and Baluchestan, Ardabil, West Azerbaijan, East Azerbaijan, Bushehr, Chaharmahal Bakhtiari, Hormozgan, Isfahan, Kerman, Khorasan Razavi, Kohkiluyeh and Boyer Ahmad, Kurdistan, and Tehran), the LRI burden decreased. More details are provided in Table S10.

**Figure 5:**
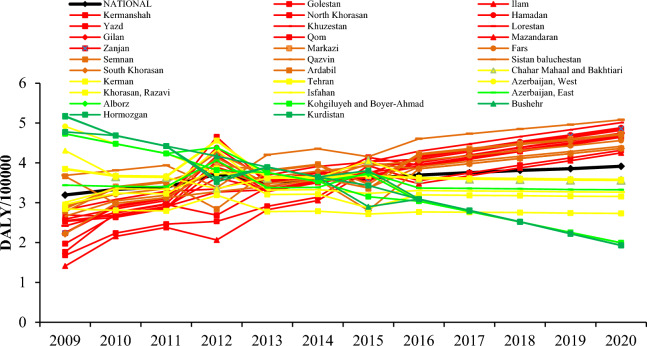
DALYs due to otitis media in relation to exposure to SHS in Iran adolescents at the provincial level in 2009–2020.

According to Fig. 5, at the provincial level, the trend of otitis media burden in 19 provinces of Iran is increasing (Ardabil, Fars, Gilan, Golestan, Hamadan, Ilam, Kermanshah, North Khorasan, South Khorasan, Khuzestan, Lorestan, Markazi, Mazandaran, Qazvin, Qom, Semnan, Sistan and Baluchestan, Yazd, Zanjan). In 10 provinces (Alborz, West Azerbaijan, East Azerbaijan, Bushehr, Chaharmahal Bakhtiari, Hormozgan, Kerman, Kohkiluyeh and Boyer Ahmad, Kurdistan, Tehran) was decreased and in two provinces of Isfahan and Khorasan Razavi was almost uniform. More details are provided in Table S11.

**Figure 6:**
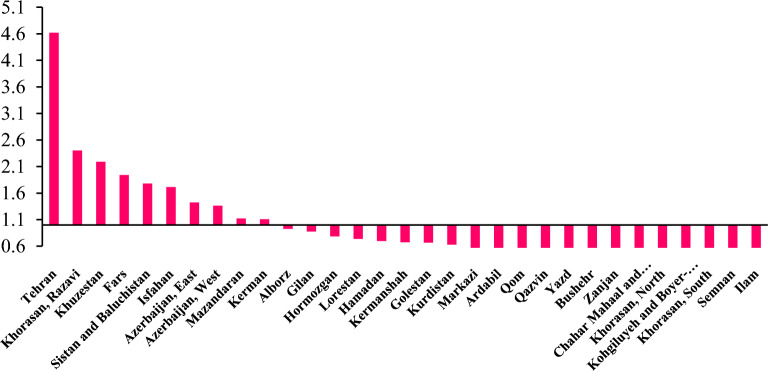
DALYs due to asthma, LRI, and otitis media in relation to exposure to SHS in Iran adolescents at the provincial level compared to the national average DALYs.

According to Fig. 6, the highest DALY of asthma, LRI and otitis media attributed to exposure to SHS is related to the provinces of Tehran, Khorasan Razavi and Khuzestan, respectively compared to the national average. In addition, the lowest DALY was related to Ilam, Semnan, and South Khorasan, respectively.

**Figure 7:**
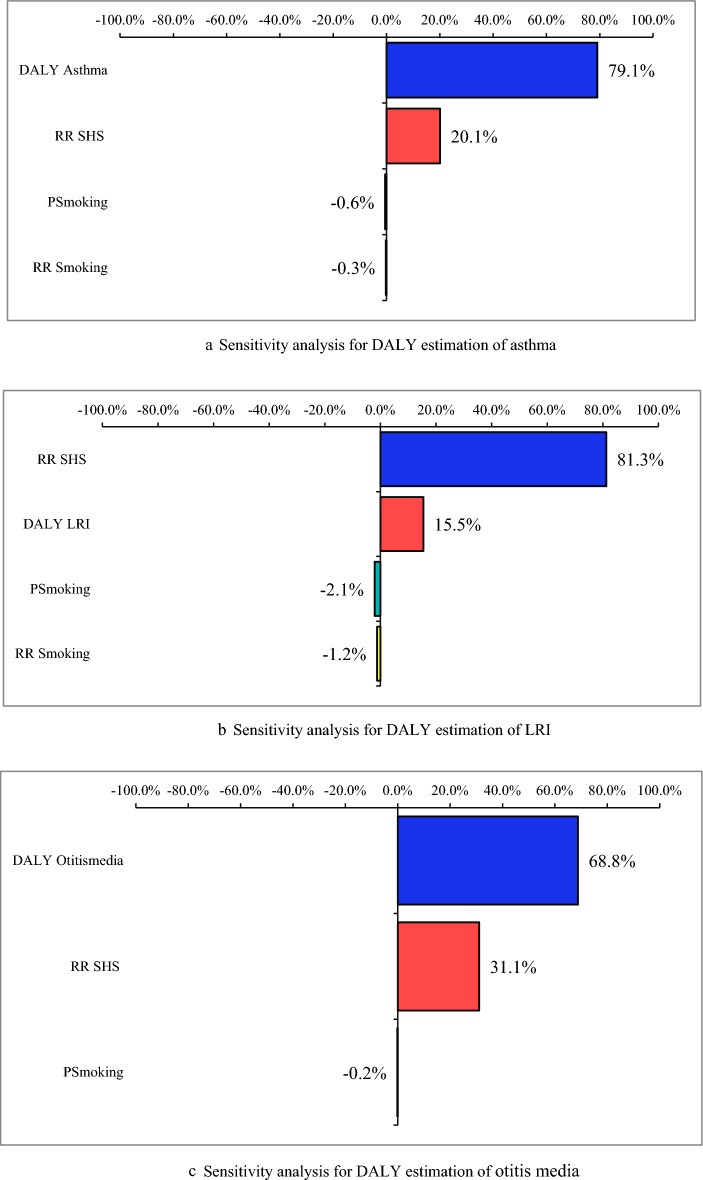
(**a**-**c**) Sensitivity analysis results for DALYs estimation of selected outcomes attributed to SHS exposure.

Figure 7a–c show the sensitivity analysis results for DALYs estimation of selected outcomes attributed to SHS exposure. Sensitivity analysis showed that the variation in the Iran burden of diseases had the greatest effect on the dispersion of the results**,** respectively for asthma (79.1%), and otitis media (68.8%), then, SHS relative risk for LRI (81.3%) DALY estimation had the greatest effect on the dispersion of the results.

## Discussion

SHS is still known as one of the most common indoor pollutants worldwide. The results of the present study suggested the high rate of population exposure to SHS in Iran. Based on national Caspian studies in Iran (which enjoy a common study design), the prevalence of exposure to SHS among adolescents was reported 48.5, 43.87, and 44% in 2010, 2012, and 2015, respectively^15,20,21^. Based on the present study, data for exposure assessment is limited, and it seems essential to pay more attention to SHS exposure in conducting national surveys and obtain sufficient and accurate information in different age groups and the national and sub-national levels for accurate estimates. The study also indicated that most studies did not consider the other tobacco products exposure, such as hookah, as the practical definition of SHS exposure in their research, while the prevalence of exposure to other tobacco products is also common. In addition, factors such as places of exposure, duration of exposure, or frequency of exposure were not considered in many studies. Therefore, it seems more effective to ask more detailed questions to gain more information about SHS exposure and gather more accurate information.

According to the present study results, the highest burden of diseases in Iran was related to LRI, asthma, and otitis media, respectively. The trends in disease burden showed that asthma, LRI, and otitis media burdens slightly decreased from 2009 to 2020. The results of studies justified the declining trend in LRI with successful international efforts such as the Millennium Development Goals (MDGs) in reducing disease burden. The decline acted differently in various countries, so that in some developing countries, the mortality rate was much higher than the rates set by international targets. In contrast, according to the Global Burden of Disease study (GBD), the reduction rate was much lower in other developing countries. The trend of diseases was different in different countries; in some countries, the mortality rate decreased but the incidence showed an increase. In these countries, threatening behaviors such as insufficient handwashing with soap or exposure to SHS decreased. According to this study, the strategies to reduce lower respiratory infections could not be generalized as they were found to be different for each specific region; as a result, it seems essential to make certain adjustments in the field of appropriate interventions^22^. Despite advances in asthma treatment in recent decades, development is still necessary for disease prevention and patient improvement^23^. According to studies, anyone can develop asthma, but the risk is very low without a risk factor. Although the genetic predisposition to asthma is quite obvious, the gene-environment interaction may explain many global changes in asthma and allergies^24^. Major risk factors for asthma include lifestyle factors, occupational exposure, allergens, tobacco smoke, and outdoor and indoor pollution^25,26^. Zonglin He et al. studied the association between secondhand smoke and childhood asthma by a systematic review and meta-analysis, based on this study significantly positive associations were observed between SHS exposure and asthma-like syndrome (OR 1.34; 95% CI 1.34–1.64), wheezing (OR 1.27; 95% CI 1.23–1.32) and doctor-diagnosed asthma (OR 1.24; 95% (CI) 1.20–1.28)^27^. In other studies, also SHS exposure considered as a modifiable risk factor associated with childhood and adult asthma^28^. According to studies, the prevalence of otitis media may also vary due to geographical differences and different study designs. Although the vaccination causes a reduction in otitis media in many areas, its rate is still high in many countries^29^. Poor living conditions, exposure to SHS, and lack of access to medical care are all significant risk factors for otitis media. Appropriate interventions are recommended to prevent or recover patients^30,31^.

The results showed that the national average asthma burden (DALY/100,000) attributed to SHS exposure in 2009 increased from 17.4 (11.8_23.9) to 21.3 (13.9_30) 2020 in Iran. Continente et al.^32^ reported that 140,000 incident cases of respiratory health outcomes in Spainish children were attributable to SHS exposure in 2015. Exposure or susceptibility to internal pollutants, including tobacco smoke, air pollutants, and allergens, are three important risk factors for asthma. Although the role of indoor exposure in the development or exacerbation of asthma is mainly unknown, strong evidence suggests that indoor exposure factors play a key role in stimulating and exacerbating asthma, allergy, and respiratory symptoms^33^. Based on the results of the present study, the increasing trend of asthma burden is justified by the increasing trend of exposure to SHS^12^. The results are consistent with other studies that confirmed the role of SHS exposure in the prevalence of the disease and the exacerbation of asthma symptoms. Also, tobacco smoke, including exposure to SHS, is a common trigger for asthma^34–37^. Korsbæk et al.^38^, considered the risk of asthma in 20,421 adults exposed to SHS at different stages of life. Based on this study 12% had been lifelong exposed to SHS, 2% and 69% had been exposed in adulthood and childhood respectively. Corresponding odds ratios for asthma reported as 1.36 (1.14–1.63), 1.49 (1.09–2.05), and 1.13 (0.99–1.30), respectively, compared with non-exposed individuals^38^.

According to the present study, the national average burden of otitis media (DALY/100,000) attributed to SHS exposure in 2009 increased from 3.1(1.9_4.6) to 3.9(2.4_5.6) in 2020. A review of studies showed that parental smoking increased the risk of otitis media. Csákányi et al.^39^ studied the relationship between exposure to SHS and otitis media in Hungarian children. Of the 412 participants, 155 (38%) parents of children with otitis media were smokers. In addition, parental smoking increased the risk of recurrent otitis media in children by more than double. Among children whose parents smoked, consuming half a pack of cigarettes a day and attending day care had a two-fold or nearly three-fold increased risk of developing recurrent otitis media, respectively^39^. It has been hypothesized that SHS exposure increases the risk of otitis media via affecting the eustachian tube’s mucociliary clearance, ciliary beat frequency, or mucous hypersecretion. SHS exposure might also worsen or prolong the recovery from otitis media by inflaming the Eustachian tube^40^. Patel et al.^41^ reported that exposure to high concentrations of SHS may increase the prevalence of eustachian tube dysfunction. The odds ratios of eustachian tube dysfunction for individuals ages 12–15 exposed to SHS in the first, second, and third tertiles of cotinine concentrations were 1.38 (0.53–3.60), 0.99 (0.53–3.60), and 2.67 (1.12–6.34), respectively, compared to an unexposed individual. The corresponding levels for those ages 16–19 were 1.28 (0.48–3.41), 0.99 (0.40–2.48), and 2.86 (1.19–6.88), respectively^41^.

The national average burden of LRI (DALY/100,000) attributed to SHS exposure in 2009 decreased from 25.8 (21.5_30.2) to 19.8 (16.7_23.1) in 2020 in the present study. The trend of this disease was incrementally decreasing, while the highest incidence of the disease for 2012 was 29.1 (24.5/33.9). Even though exposure to SHS in Iran showed an increasing trend^12^, the decreasing trend of this disease was related to reducing the burden of this disease worldwide. The findings of the GBD studies indicated a significant improvement in the burden of LRI due to a reduction in major risk factors. Still, this improvement was not equivalent in different locations and might require more effort among some populations. According to these studies, most epidemiological studies are conducted in developed countries. Conditions in developing countries could be different from those in high-income countries. Factors that influence the differences in the severity of SHS exposure between developing and developed countries are as follows: the severity of smoking, natural ventilation, residential density in the house, smoke from solid fuel used for cooking, and enforce legal protection against SHS exposure in indoor workplaces and public places^14,42^.

The present study results indicated that the trends of all three outcomes burden in Iran adolescents were increasing in most provinces. The trend of asthma and otitis media burden attributed to SHS exposure increased in 61.2% of provinces, and lower respiratory infection in 41.9% of provinces. This increase in the burden of SHS in most provinces of Iran, despite the decreasing trend of the burden of these diseases worldwide, showed that the reduction of exposure to SHS in Iran has ignored. Following worldwide efforts and the enactment of laws to reduce exposure to SHS, currently, only 7.4% of the world's populations live in areas with comprehensive smoke-free regulations. According to GBD studies, effective interventions to reduce exposure to tobacco smoke were performed in public and private places in many countries. By the end of 2007, 16 countries had enacted national tobacco-free laws that covered all high-risk environments, including workplaces and public places. To evaluate the effectiveness of this type of law, exposure to SHS in high-risk environments (such as bars and restaurants) was typically reduced by about 90%, and exposure to non-smokers in the population was reduced by up to 60%^14^. Other reports on the implementation of smoking bans showed the effectiveness of these laws^43^.

Uncertainty assessment in this study showed that uncertainties included: exposure data (gaps in exposure prevalence data that were completed by statistical modeling), selection of the study population, active smoking burden of diseases, the total disease burden, and the relative risk of health outcomes, which were reported in other similar studies^14^. Sensitivity analysis showed that the Sensitivity analysis showed that the variation in global DALYs and relative risk had the greatest effect on the dispersion of the results. The total burden of diseases and relative risk that were taken from world-class studies, also, the lack of uniform definition of exposure caused uncertainty. Considering the same definitions and paying attention to the details of exposure, information provided by global organizations to achieve uniform data with less uncertainty can be useful for estimates that are more accurate. In addition, with the rapid increase in electronic cigarette users worldwide, considering the electronic cigarette consumption pattern and environmental exposure to these kinds of cigarettes in studies can provide a more comprehensive Figure of the impacts of smoking habits in more detail.

## Limitations

This study offered implications, including variations in definitions of SHS exposure across available studies, which were included in the systematic review, and we used the national surveys to obtain uniform data. Lack of complete smoking data and SHS exposure data by age and sex in all provinces of Iran from 2009 to 2020 made us predict data through modeling. Also, robust global data on the global burden of diseases was used for Iran's burden of diseases instead of national data.

## Conclusion

There is limited information on exposure to SHS and its attributed burden of diseases in Middle Eastern countries, and most epidemiological studies were conducted in developed countries. This study considered the SHS-attributed burden of diseases in Iran to provide enough evidence for health policy and planning. The study showed the high DALY rate attributed to SHS exposure in Iran. The detailed results showed the increasing DALY rate of asthma and otitis media attributed to SHS exposure. Also, the trend of LRI was also increasing and then somewhat decreasing in Iran during the study period. The increasing trend of SHS-attributable DALYs in Iran results from the increasing trend of SHS exposure. Reducing SHS exposure needs more attention from policymakers to protect the population. Smoke-free laws prohibiting smoking in public places, supporting the creation of non-smoking venues, providing knowledge for keeping physical distance from the smoker, and providing smoke-free homes and personal cars are recommended.

### Supplementary Information

Supplementary Information.

## Data Availability

The datasets used in the present study are available from the corresponding author on reasonable request.
