# Supplementary material for: Burden of diseases attributable to second-hand smoke exposure in Iran adolescents from 2009 to 2020
Source: Sci Rep. 2023 Aug 21;13:13605. doi: 10.1038/s41598-023-40058-z (PMC10442427; doi:10.1038/s41598-023-40058-z)
Supplement: Supplementary file 1 — Supplementary Information. [file 41598_2023_40058_MOESM1_ESM.docx]

**Burden of Diseases Attributable to Second-hand Smoke Exposure in Iran Adolescents from 2009 to 2020**

Hosna Janjani^1^, Ramin Nabizadeh^1^, Mansour Shamsipour ^2,3^, Homa Kashani^2^, Mina Aghaei^1^, Masud Yunesian^1,2^

^1^ *Department of Environmental Health Engineering, School of Public Health, Tehran University of Medical Sciences, Tehran, Iran*

*^2^ Department of Research Methodology and Data Analysis, Institute for Environmental Research, Tehran University of Medical Sciences, Tehran, Iran*

*^3^Center for Air Pollution Research (CAPR), Institute for Environmental Research (IER), Tehran University of Medical Sciences, Tehran, Iran*

Table.S1.Iran's provinces by area and population

| Province (Abbreviation) | Area (km^2^( | Population |
| --- | --- | --- |
| Alborz (AL ) | 5122 | 2712400 |
| Ardabil (AR ) | 17800 | 1270420 |
| Azarbaijan East (EA) | 45651 | 3909652 |
| Azarbaijan West (WA ) | 37411 | 3265219 |
| Bushehr (BS) | 27743 | 1163400 |
| Chahar mahal and Bakhtiari (CM) | 16328 | 947763 |
| Fars (FA) | 122608 | 4851274 |
| Gilan (GI) | 14042 | 2530696 |
| Golestan (GO) | 20367 | 1868619 |
| Hamadan (HD) | 19368 | 1738214 |
| Hormozgan (HG) | 70679 | 1776415 |
| Ilam (IL) | 20133 | 580158 |
| Isfahan (ES ) | 107018 | 5120850 |
| Kerman(KE) | 183193 | 516718 |
| Kermanshah (BK) | 24998 | 1952434 |
| khorasan north (KS) | 28434 | 863092 |
| Khorasan Razavi (KV) | 118018 | 6434501 |
| Khorasan south (KJ) | 151193 | 768898 |
| Khuzestan (KZ) | 64055 | 4710509 |
| Kohgiluyeh and boyer Ahmad (KB) | 15504 | 713052 |
| Kordestan (KD) | 29137 | 1603011 |
| Lorestan ( LO) | 28294 | 1760649 |
| Markazi (MK) | 29127 | 1429475 |
| Mazandaran (MN) | 23842 | 3283582 |
| Qazvin (QZ) | 15567 | 1273761 |
| Qom(QM) | 11562 | 1201158 |
| Semnan (SM ) | 97491 | 70236 |
| Sistan Baluchestan (SB ) | 180726 | 2775049 |
| Tehran (TE ) | 13692 | 13267637 |
| Yazd (YA) | 73477 | 1138533 |
| Zanjan (ZA) | 21773 | 1057461 |


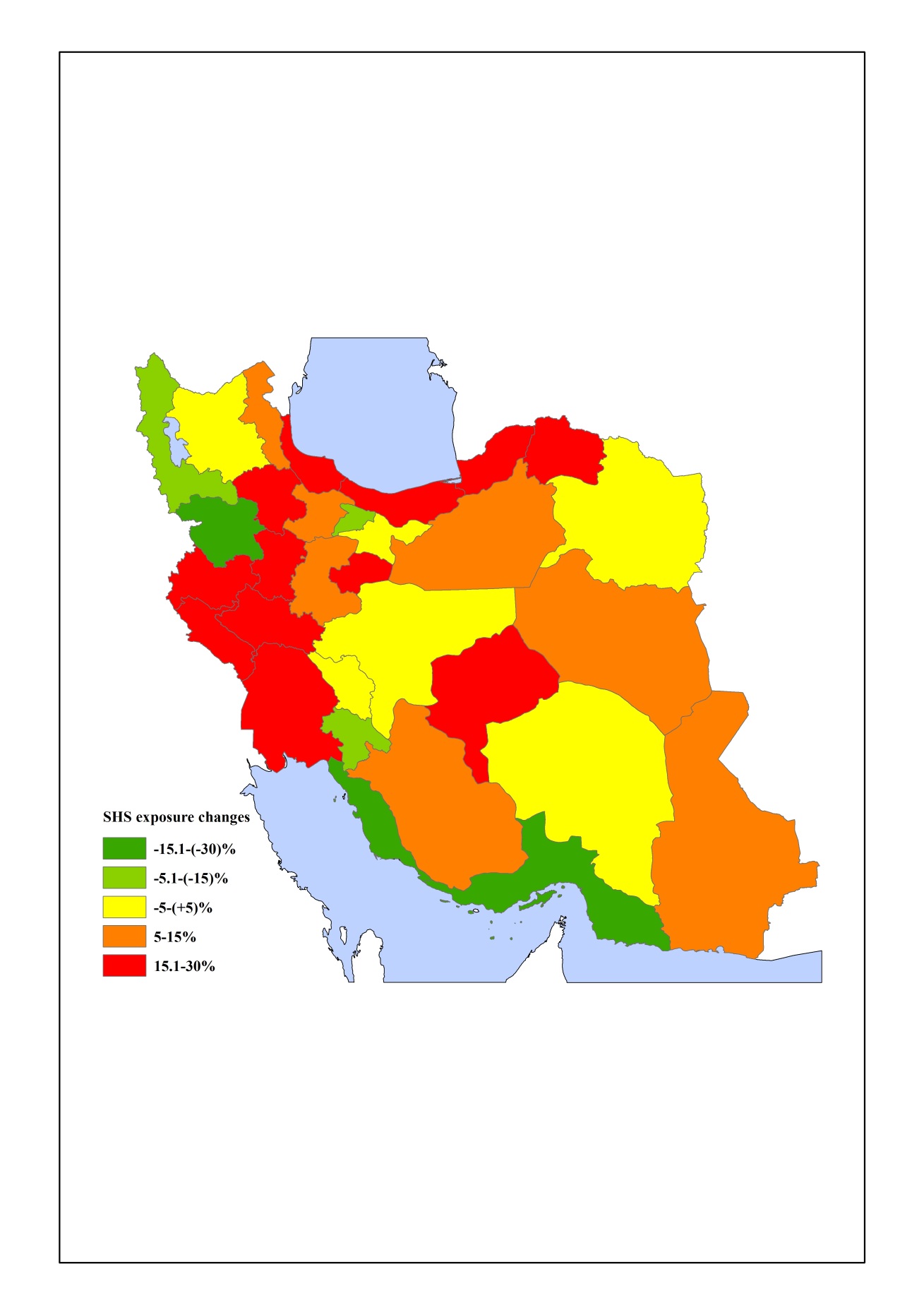


Figure s1. Classification of provinces based on changes in exposure to SHS 2009-2015(Map created using ArcMap 10.3, (http://desktop.arcgis.com/en/arcmap/)

TableS2. Components of Variance

| **Extraction Sums of Squared Loadings** | | **Rotation Sums of Squared Loadings** | | **Initial Eigenvalues** | | |  |
| --- | --- | --- | --- | --- | --- | --- | --- |
| Cumulative % | % of Variance | Cumulative % | % of Variance | | Cumulative % | % of Variance |  |
| 38.031 | 38.031 | 31.159 | 31.519 | | 38.031 | 38.031 | 1 |
| 57.561 | 19.530 | 55.354 | 24.195 | | 57.561 | 19.530 | 2 |
| 57.114 | 17.553 | 75.114 | 19.760 | | 75.114 | 17.553 | 3 |
|  |  |  |  | | 84.139 | 9.524 | 4 |
|  |  |  |  | | 91.818 | 7.180 | 5 |
|  |  |  |  | | 96.980 | 5.162 | 6 |
|  |  |  |  | | 100.000 | 3.020 | 7 |

Table S3. Component matrix

| **Component3** | **Component2** | **Component1** | **Variable** |
| --- | --- | --- | --- |
| 0.806 | 0.103 | -0.170 | **Development Index** |
|  | 0.597 | 0.612 | **The cost of tobacco products** |
|  | 0.875 | 0.101 | **smoking** |
| 0.118 | 0.738 | 0.290 | **Urbanization** |
| 0.102 |  | 0.923 | **Household expenses** |
|  |  | 0.904 | **Household income** |
| 0.833 |  | -0.116 | **Employment** |

**Table S4.** Regression Analysis for Variables Predicting SHS

| **P** | **t** | **Beta** | **SE** | **B** | **Model** |  |
| --- | --- | --- | --- | --- | --- | --- |
| 0.000 | 15.410 | -0.651 | 4.235 | 65.262 | (Constant) |  |
| 0.001 | -3.682 | 0.213 | 0.976 | -3.594 | year |  |
| 0.369 | 0.919 | 0.090 | 2.657 | 2.442 | REGR factorscore 2foranalysis 1 | Sharp decrease |
| 0.708 | 0.379 |  | 4.082 | 1.549 | REGR factor score 3 for analysis 1 |  |
| 0.000 | 10.813 | -0.655 | 5.683 | 61.447 | (Constant) | Mild decrease |
| 0.015 | -2.829 | -.011 | 1.135 | -3.210 | year |  |
| 0.963 | -0.047 |  | 4.046 | -.191 | REGR factorscore 3foranalysis 1 |  |
| 0.000 | 12.882 | 0.220 | 2.924 | 37.662 | (Constant) |  |
| 0 .101 | 1.671 | -0.153 | .569 | 0.951 | year |  |
| 0.320 | -1.006 | -0.215 | 1.765 | -1.776 | REGR factor score 2foranalysis 1 | No change |
| 0.109 | -1.635 | 0.377 | 1.152 | -1.883 | REGR factor score 3 for analysis 1 |  |
| 0.016 | 2.490 |  | 1.323 | 3.295 | REGR factor score 1for analysis 1 |  |
| 0.000 | 10.720 | 0.549 | 2.843 | 30.475 | (Constant) |  |
| 0.000 | 5.008 | -0.192 | .409 | 2.047 | year |  |
| 0.179 | -1.371 | -0.350 | 1.607 | -2.204 | REGR factorscore 2foranalysis 1 | Mild increase |
| 0.042 | -2.102 | 0.237 | 2.715 | -5.707 | REGR factor score 3 for analysis 1 |  |
| 0.088 | 1.753 |  | 1.291 | 2.263 | REGR factor score 1for analysis 1 |  |
| 0.000 | 14.094 | 0.644 | 1.725 | 24.312 | (Constant) |  |
| 0.000 | 8.749 | 0.187 | 0.333 | 2.911 | year | Sharp increase |
| 0.022 | 2.335 | 0.192 | 1.234 | 2.882 | REGR factorscore 2foranalysis 1 |  |
| 0.041 | 2.076 | .1830 | 1.149 | 2.385 | REGR factor score 3 for analysis 1 |  |
| .0410 | 2.074 | -0.651 | 1.567 | 3.250 | REGR factor score 1for analysis 1 |  |

TableS5. Coefficient of Determination of Regression Analysis for Variables Predicting SHS

| **STD** | **ADJ R^2^** | **R^2^** | **R** |  |
| --- | --- | --- | --- | --- |
| 10.758 | 0.336 | 0.422 | 0.650 | **Sharp decrease** |
| 9.323 | 0.329 | 0.425 | 0.652 | **Mild decrease** |
| 9.722 | 0.146 | 0.215 | 0.463 | **No change** |
| 6.238 | 0.523 | 0.569 | 0.755 | **Mild increase** |
| 8.107 | 0.469 | 0.491 | 0.700 | **Sharp increase** |

TableS6. Regression Analysis for Predicting DALY values for 2020

| R^2^ | Regression model |  | Age group | Health outcome |
| --- | --- | --- | --- | --- |
| 0.84 | y= -0.6138x + 174.33 | Mean | 10-19 | Asthma |
| 0.37 | y= -0.4912x + 267.85 | Upper limit |  |  |
| 0.96 | y= -1.166x + 110.87 | Lower limit |  |  |
| 0.96 | y= -0.0808x + 30.426 | Mean | 10-19 | Otitis media |
| 0.88 | y= -0.1385x + 50.873 | Upper limit |  |  |
| 0.68 | y= -0.0333x + 16.966 | Lower limit |  |  |
| 0.91 | y= -7.1037x + 195.03 | Mean | 10-19 | LRI |
| 0.92 | y= -7.565x + 211.96 | Upper limit |  |  |
| 0.92 | y= -6.2522x + 173.94 | Lower limit |  |  |

TableS7. DALYs of asthma, otitis media, and LRI in Iran adolescents 2009-2020

| **DALY/100000** | | | **Year** |
| --- | --- | --- | --- |
| **Asthma** | **LRI** | **Otitis media** |  |
| 174.71(109.65 -271.53) | 178.29(159.17 -196.03) | 30.41(17.01 -51) | 2009 |
| 173.05(108.06 -269.42) | 175.06(157.13 -191.47) | 30.25(16.83 -50.6) | 2010 |
| 172(107.28 -268.33) | 173.7(155.16 -188.57) | 30.15(16.81 -50.33) | 2011 |
| 171.79(106.34 -268.76) | 173.97(154.88 -188.14) | 30.05(16.88 -50.07) | 2012 |
| 171.8(106.06 -269.03) | 174.35(154.83 -189.81) | 29.98(16.77 -49.94) | 2013 |
| 170.36(104.98 -269.62) | 161.4(144.19 -175.04) | 29.92(16.86 -50.17) | 2014 |
| 168.08(102.02 -267.39) | 142.37(127.8 -154.93) | 29.87(16.59 -49.91) | 2015 |
| 168.63(100.12 -271.57) | 132.55(119.26 -144.98) | 29.82(16.64 -49.94) | 2016 |
| 169.82(100.22 -274.67) | 128.12(115.88 -140.91) | 29.77(16.73 -49.56) | 2017 |
| 168.93(99.4 -275.47) | 120.92(108.95 -133.74) | 29.64(16.6 -49.61) | 2018 |
| 167.82(98.46 -272.89) | 115.64(103.38 -128.61) | 29.45(16.65 -49.27) | 2019 |
| 166.96(96.87 -273.74) | 109.78(98.91 -121.16) | 29.45(16.56 -49.21) | 2020 |

Table S8. DALYs of asthma, otitis media, and LRI attributable to SHS exposure in Iran adolescents 2009-2020

| **DALY/100000** | | | **Year** |
| --- | --- | --- | --- |
| **Asthma** | **LRI** | **Otitis media** |  |
| 17.4(11.8_23.9) | 25.8(21.5_30.2) | 3.1(1.9_4.6) | 2009 |
| 18.1(12.3_24.9) | 26.5(22.2_31) | 3.3(2_4.8) | 2010 |
| 18.3(12.4_25.3) | 26.7(22.3_31.1) | 3.3(2_4.9) | 2011 |
| 20.2(13.7_27.8) | 29.1(24.5_33.9) | 3.7(2.3_5.4) | 2012 |
| 19.1(12.9_26.3) | 27.8(23.3_32.4) | 3.5(2.1_5.1) | 2013 |
| 19.4(13.1_26.8) | 26.2(22_30.5) | 3.5(2.2_5.2) | 2014 |
| 19.4(13_27) | 23.5(19.7_27.4) | 3.6(2.2_5.2) | 2015 |
| 19.9(13.2_27.9) | 22.4(18.8_26) | 3.6(2.2_5.3) | 2016 |
| 20.5(13.5_28.7) | 22.1(18.5_25.7) | 3.7(2.3_5.4) | 2017 |
| 20.8(13.6_29.2) | 21.2(17.8_24.7) | 3.8(2.3_5.5) | 2018 |
| 21(13.8_29.4) | 20.6(17.2_24) | 3.8(2.3_5.6) | 2019 |
| 21.3(13.9_30) | 19.8(16.7_23.1) | 3.9(2.4_5.6) | 2020 |

**Table S9.** Burden of asthma (DALY/100000) related to SHS exposure in Iran adolescents at the provincial level (2009-2020)

| **2020** | **2019** | **2018** | **2017** | **2016** | **2015** | **2014** | **2013** | **2012** | **2011** | **2010** | **2009** | **Province** |
| --- | --- | --- | --- | --- | --- | --- | --- | --- | --- | --- | --- | --- |
| 21.3 (13.9_30) | 21 (13.8_29.4) | 20.8 (13.6_29.2) | 20.5 (13.5_28.7) | 19.9 (13.2_27.9) | 19.4 (13_27) | 19.4 (13.1_26.8) | 19.1 (12.9_26.3) | 20.2 (13.7_27.8) | 18.3 (12.4_25.3) | 18.1 (12.3_24.9) | 17.4 (11.8_23.9) | National |
| 23.4 (15.3_33) | 22.4 (14.7_31.5) | 21.5 (14.1_30.3) | 20.4 (13.4_28.6) | 19.1 (12.6_26.8) | 21.1 (14.1_29.3) | 17 (11.4_23.5) | 15.8 (10.7_21.9) | 13.7 (9.3_19) | 13.3 (9_18.4) | 12.1 (8.2_16.7) | 9.1 (6.1_12.6) | Golestan |
| 23.1 (15_32.5) | 22.1 (14.5_31) | 21.1 (13.9_29.7) | 20 (13.2_28.1) | 18.7 (12.4_26.2) | 20.4 (13.6_28.3) | 16.5 (11.1_22.9) | 15.3 (10.3_21.2) | 11.2 (7.5_15.5) | 12.9 (8.7_17.8) | 11.7 (7.9_16.1) | 7.6 (5.1_10.6) | Ilam |
| 25.5 (16.6_35.8) | 24.5 (16.1_34.4) | 23.6 (15.5_33.2) | 22.6 (14.9_31.6) | 21.3 (14.1_29.7) | 22.2 (14.9_30.8) | 19.2 (12.9_26.6) | 18.1 (12.2_25) | 17.7 (12_24.5) | 15.7 (10.6_21.7) | 14.6 (9.8_20.1) | 10.7 (7.2_14.8) | Kermanshah |
| 25.7 (16.8_36.1) | 24.8 (16.3_34.7) | 23.9 (15.7_33.5) | 22.9 (15.1_32) | 21.6 (14.3_30.2) | 19.4 (13_27) | 19.5 (13.1_27) | 18.4 (12.4_25.4) | 14.5 (9.8_20.1) | 16 (10.8_22.1) | 14.9 (10.1_20.5) | 9.6 (6.5_13.2) | Khorasan, North |
| 26.5 (17.4_37.3) | 25.6 (16.8_35.8) | 24.7 (16.3_34.7) | 23.7 (15.7_33.2) | 22.5 (14.9_31.4) | 20.1 (13.4_27.9) | 20.4 (13.8_28.3) | 19.3 (13.1_26.7) | 23.8 (16.1_32.7) | 17 (11.5_23.4) | 15.8 (10.7_21.8) | 12.2 (8.2_16.8) | Hamadan |
| 26.2 (17.1_36.8) | 25.2 (16.6_35.3) | 24.4 (16_34.2) | 23.4 (15.4_32.7) | 22.1 (14.6_30.8) | 21.6 (14.5_30) | 20 (13.4_27.7) | 18.9 (12.8_26.1) | 25.3 (17.2_34.8) | 16.6 (11.2_22.8) | 15.4 (10.4_21.2) | 14 (9.5_19.4) | Yazd |
| 27.2 (17.8_38.3) | 26.3 (17.3_36.8) | 25.4 (16.7_35.7) | 24.4 (16.1_34.1) | 23.2 (15.3_32.3) | 21.6 (14.4_30) | 21.2 (14.3_29.3) | 20.1 (13.6_27.7) | 18.1 (12.3_25) | 17.8 (12.1_24.5) | 16.7 (11.3_23) | 15.3 (10.4_21.1) | Khuzestan |
| 25.8 (16.9_36.3) | 24.8 (16.3_34.8) | 24 (15.8_33.6) | 22.9 (15.1_32) | 21.6 (14.3_30.2) | 19.4 (13_26.9) | 19.5 (13.1_27) | 18.5 (12.5_25.5) | 22.7 (15.4_31.2) | 16.1 (10.9_22.2) | 14.9 (10.1_20.6) | 13.1 (8.9_18.1) | Lorestan |
| 25.3 (16.6_35.6) | 24.4 (16_34.1) | 23.5 (15.5_33) | 22.4 (14.8_31.4) | 21.1 (14_29.5) | 20.2 (13.5_28) | 19 (12.8_26.4) | 17.9 (12.1_24.8) | 22 (14.9_30.2) | 15.5 (10.5_21.5) | 14.4 (9.7_19.9) | 14.6 (9.9_20.1) | Gilan |
| 25.2 (16.5_35.5) | 24.3 (15.9_34) | 23.4 (15.4_32.9) | 22.3 (14.7_31.3) | 21.1 (13.9_29.4) | 19.8 (13.3_27.5) | 18.9 (12.7_26.2) | 17.8 (12.1_24.6) | 20.9 (14.1_28.8) | 15.4 (10.4_21.3) | 14.3 (9.6_19.7) | 13.7 (9.3_18.9) | QOM |
| 25.5 (16.6_35.8) | 24.5 (16.1_34.4) | 23.6 (15.5_33.2) | 22.6 (14.9_31.6) | 21.3 (14.1_29.8) | 21.3 (14.3_29.6) | 19.2 (12.9_26.7) | 18.1 (12.3_25) | 19.7 (13.3_27.1) | 15.7 (10.6_21.7) | 14.5 (9.9_20.1) | 13.8 (9.4_19.1) | Mazandaran |
| 26.4 (17.3_37.1) | 25.4 (16.7_35.6) | 24.6 (16.2_34.5) | 23.6 (15.6_33) | 22.3 (14.8_31.2) | 19 (12.7_26.4) | 20.3 (13.6_28) | 19.1 (12.9_26.4) | 21.1 (14.3_29.1) | 16.8 (11.4_23.1) | 15.7 (10.6_21.6) | 12.1 (8.2_16.7) | Zanjan |
| 25.7 (16.8_36.1) | 25 (16.5_35.1) | 24.5 (16.1_34.4) | 23.8 (15.7_33.3) | 22.8 (15.1_31.8) | 18.6 (12.4_25.8) | 21.5 (14.4_29.7) | 20.8 (14_28.6) | 21.4 (14.5_29.4) | 19.2 (13_26.4) | 18.4 (12.5_25.4) | 15.5 (10.5_21.3) | Markazi |
| 24.8 (16.2_34.9) | 24.1 (15.8_33.8) | 23.6 (15.5_33.1) | 22.8 (15.1_31.9) | 21.9 (14.5_30.5) | 21.1 (14.1_29.2) | 20.5 (13.8_28.3) | 19.8 (13.4_27.3) | 23.1 (15.7_31.8) | 18.1 (12.3_25) | 17.4 (11.8_23.9) | 15.9 (10.8_21.9) | Fars |
| 24 (15.7_33.8) | 23.3 (15.3_32.7) | 22.8 (14.9_32) | 22 (14.5_30.9) | 21.1 (13.9_29.4) | 18.1 (12_25.1) | 19.6 (13.2_27.1) | 18.9 (12.8_26.1) | 22.4 (15.2_30.9) | 17.3 (11.7_23.8) | 16.5 (11.2_22.7) | 14.4 (9.8_19.9) | Qazvin |
| 25.6 (16.7_36) | 24.9 (16.4_34.9) | 24.4 (16_34.1) | 23.6 (15.6_33.1) | 22.7 (15_31.7) | 18 (12_25) | 21.3 (14.4_29.5) | 20.6 (13.9_28.4) | 15.4 (10.4_21.3) | 19 (12.9_26.2) | 18.3 (12.4_25.2) | 15.1 (10.2_20.8) | Semnan |
| 27.6 (18.1_38.8) | 27 (17.8_37.8) | 26.5 (17.4_37.1) | 25.8 (17.1_36.1) | 24.8 (16.5_34.7) | 22.3 (14.9_31) | 23.6 (15.9_32.5) | 22.9 (15.5_31.5) | 18.3 (12.4_25.3) | 21.3 (14.5_29.3) | 20.7 (14_28.4) | 20 (13.6_27.5) | Sistan and Baluchistan |
| 23.7 (15.5_33.4) | 23 (15.2_32.3) | 22.5 (14.7_31.5) | 21.7 (14.3_30.4) | 20.8 (13.7_29.1) | 15 (10_21) | 19.3 (13_26.8) | 18.6 (12.5_25.6) | 17.8 (12_24.5) | 16.9 (11.5_23.3) | 16.2 (11_22.3) | 12 (8.2_16.6) | Khorasan, South |
| 23.7 (15.5_33.4) | 23 (15.1_32.3) | 22.5 (14.8_31.5) | 21.7 (14.3_30.4) | 20.8 (13.7_29) | 21.3 (14.2_29.5) | 19.3 (13_26.8) | 18.6 (12.5_25.7) | 20.9 (14.2_28.8) | 16.9 (11.5_23.3) | 16.2 (11_22.3) | 19.9 (13.5_27.3) | Ardabil |
| 17.7 (11.5_25.1) | 17.8 (11.6_25) | 17.9 (11.8_25.2) | 18 (11.8_25.2) | 17.8 (11.7_25) | 17.3 (11.6_24.1) | 18 (12.1_24.9) | 18 (12.2_24.9) | 23.5 (15.9_32.3) | 18.1 (12.3_24.9) | 18.2 (12.4_25) | 16.3 (11.1_22.5) | Isfahan |
| 14.8 (9.6_21) | 14.9 (9.7_21) | 15 (9.8_21.2) | 15 (9.9_21.1) | 14.9 (9.8_20.9) | 14.5 (9.7_20.3) | 15.1 (10.1_20.9) | 15.1 (10.2_20.9) | 17.3 (11.7_23.9) | 15.1 (10.2_20.8) | 15.2 (10.3_20.9) | 15.4 (10.5_21.3) | Kerman |
| 19.4 (12.7_27.4) | 19.5 (12.8_27.4) | 19.7 (12.9_27.7) | 19.7 (13_27.6) | 19.5 (12.9_27.3) | 19.7 (13.2_27.4) | 19.7 (13.3_27.3) | 19.8 (13.4_27.3) | 24.7 (16.8_34) | 19.8 (13.5_27.2) | 19.9 (13.5_27.4) | 20.9 (14.3_28.7) | Tehran |
| 18.1 (11.8_25.5) | 18.1 (11.9_25.5) | 18.3 (12_25.7) | 18.3 (12_25.7) | 18.2 (12_25.4) | 20.2 (13.5_28) | 18.3 (12.3_25.4) | 18.4 (12.4_25.3) | 22 (14.9_30.3) | 18.4 (12.5_25.3) | 18.5 (12.6_25.4) | 18.7 (12.7_25.6) | Azerbaijan, East |
| 17.1 (11.1_24.2) | 17.2 (11.2_24.2) | 17.3 (11.3_24.4) | 17.4 (11.4_24.3) | 17.2 (11.4_24.1) | 15.4 (10.3_21.4) | 17.4 (11.7_24.1) | 17.4 (11.8_24.1) | 21 (14.2_28.9) | 17.5 (11.8_24.1) | 17.6 (11.9_24.2) | 16 (10.9_22.1) | Khorasan, Razavi |
| 19.3 (12.6_27.3) | 19.4 (12.7_27.2) | 19.5 (12.8_27.5) | 19.6 (12.9_27.4) | 19.4 (12.8_27.2) | 21.8 (14.6_30.2) | 19.6 (13.2_27.1) | 19.6 (13.3_27.1) | 18.2 (12.3_25.1) | 19.7 (13.4_27.1) | 19.8 (13.5_27.2) | 23.4 (16_32.1) | Chahar Mahaal and Bakhtiari |
| 10.8 (7_15.3) | 12.2 (8_17.3) | 13.7 (9_19.4) | 15.2 (9.9_21.3) | 16.4 (10.8_22.9) | 16.9 (11.3_23.5) | 19.1 (12.8_26.5) | 20.4 (13.8_28.2) | 20.7 (14_28.6) | 22.9 (15.6_31.5) | 24.3 (16.6_33.3) | 25.7 (17.6_35.2) | Kohgiluyeh and Boyer-Ahmad |
| 10.8 (7_15.3) | 12.2 (8_17.3) | 13.7 (9_19.4) | 15.2 (9.9_21.3) | 16.4 (10.8_22.9) | 20.5 (13.7_28.4) | 19.1 (12.9_26.5) | 20.5 (13.8_28.2) | 20.2 (13.7_27.9) | 22.9 (15.6_31.5) | 24.3 (16.6_33.3) | 26.7 (18.3_36.6) | Azerbaijan, West |
| 10.8 (7_15.3) | 12.3 (8_17.3) | 13.7 (9_19.4) | 15.2 (9.9_21.3) | 16.4 (10.8_22.9) | 19.9 (13.3_27.7) | 19.1 (12.9_26.5) | 20.4 (13.8_28.2) | 23.8 (16.1_32.7) | 23 (15.6_31.5) | 24.3 (16.6_33.3) | 25.7 (17.6_35.2) | Alborz |
| 10.5 (6.8_14.8) | 12.1 (7.9_17.1) | 13.7 (9_19.4) | 15.3 (10_21.5) | 16.7 (11_23.3) | 15.5 (10.4_21.7) | 19.7 (13.3_27.3) | 21.2 (14.3_29.2) | 22.6 (15.3_31.1) | 24 (16.3_32.9) | 25.4 (17.4_34.9) | 28.1 (19.3_38.4) | Bushehr |
| 10.5 (6.8_14.8) | 12.1 (7.9_17.1) | 13.7 (9_19.4) | 15.3 (10_21.5) | 16.7 (11_23.3) | 20.4 (13.6_28.3) | 19.7 (13.2_27.3) | 21.2 (14.3_29.2) | 19.1 (12.9_26.3) | 24 (16.3_32.9) | 25.4 (17.3_34.9) | 28.2 (19.3_38.5) | Kurdistan |
| 10.5 (6.8_14.8) | 12.1 (7.9_17) | 13.7 (9_19.4) | 15.3 (10_21.5) | 16.7 (11_23.3) | 18.4 (12.3_25.6) | 19.7 (13.3_27.3) | 21.2 (14.3_29.2) | 19.5 (13.2_26.9) | 24 (16.3_32.9) | 25.4 (17.3_34.8) | 26 (17.8_35.5) | Hormozgan |

**Table S10.** Burden of LRI (DALY/100000) related to SHS exposure in Iran adolescents at the provincial level (2009-2020)

| **2020** | **2019** | **2018** | **2017** | **2016** | **2015** | **2014** | **2013** | **2012** | **2011** | **2010** | **2009** | **Province** |
| --- | --- | --- | --- | --- | --- | --- | --- | --- | --- | --- | --- | --- |
| 19.8 (16.7_23.1) | 20.6 (17.2_24) | 21.2 (17.8_24.7) | 22.1 (18.5_25.7) | 22.4 (18.8_26) | 23.5 (19.7_27.4) | 26.2 (22_30.5) | 27.8 (23.3_32.4) | 29.1 (24.5_33.9) | 26.7 (22.3_31.1) | 26.5 (22.2_31) | 25.8 (21.5_30.2) | National |
| 21.7 (18.3_25.1) | 21.9 (18.4_25.5) | 21.9 (18.4_25.4) | 22 (18.5_25.6) | 21.5 (18.1_25.1) | 25.4 (21.3_29.4) | 23.2 (19.3_27) | 23.3 (19.4_27.3) | 20.4 (16.9_23.9) | 19.8 (16.4_23.2) | 18.2 (15_21.4) | 14 (11.5_16.5) | Golestan |
| 21.3 (18_24.8) | 21.5 (18.1_25.1) | 21.5 (18.1_25) | 21.6 (18.2_25.2) | 21.1 (17.7_24.6) | 24.6 (20.7_28.6) | 22.6 (18.8_26.4) | 22.7 (18.9_26.6) | 16.7 (13.8_19.7) | 19.2 (15.9_22.5) | 17.6 (14.5_20.7) | 11.8 (9.7_14) | Ilam |
| 23.3 (19.7_27) | 23.7 (20_27.5) | 23.8 (20.1_27.6) | 24.2 (20.3_28) | 23.7 (20_27.6) | 26.6 (22.4_30.8) | 26 (21.7_30.2) | 26.5 (22.1_30.9) | 25.9 (21.6_30.2) | 23.1 (19.2_27) | 21.7 (18_25.4) | 16.3 (13.4_19.3) | Kermanshah |
| 23.6 (19.9_27.2) | 23.9 (20.2_27.8) | 24.1 (20.3_27.9) | 24.4 (20.6_28.3) | 24.1 (20.2_27.9) | 23.5 (19.7_27.4) | 26.4 (22.1_30.7) | 26.9 (22.5_31.4) | 21.5 (17.9_25.2) | 23.5 (19.6_27.5) | 22.1 (18.4_25.9) | 14.7 (12.1_17.4) | Khorasan, North |
| 24.2 (20.5_28) | 24.6 (20.8_28.6) | 24.8 (20.9_28.8) | 25.2 (21.3_29.2) | 24.9 (21_28.9) | 24.2 (20.3_28.1) | 27.5 (23.1_31.9) | 28.1 (23.6_32.8) | 33.9 (28.6_39.2) | 24.8 (20.7_29) | 23.4 (19.5_27.4) | 18.5 (15.3_21.8) | Hamadan |
| 23.9 (20.3_27.6) | 24.3 (20.5_28.2) | 24.5 (20.7_28.4) | 24.9 (21_28.8) | 24.6 (20.7_28.5) | 25.9 (21.8_30.1) | 26.9 (22.6_31.3) | 27.6 (23_32.1) | 35.8 (30.3_41.3) | 24.2 (20.2_28.3) | 22.9 (19_26.8) | 21.1 (17.5_24.8) | Yazd |
| 24.8 (21_28.6) | 25.3 (21.3_29.2) | 25.5 (21.5_29.4) | 25.9 (21.9_30) | 25.7 (21.6_29.7) | 25.9 (21.8_30.1) | 28.4 (23.9_33) | 29.2 (24.4_34) | 26.4 (22.1_30.8) | 25.9 (21.7_30.3) | 24.6 (20.5_28.8) | 22.9 (19_26.9) | Khuzestan |
| 23.6 (20_27.3) | 24 (20.2_27.8) | 24.1 (20.3_28) | 24.5 (20.6_28.4) | 24.1 (20.3_28) | 23.5 (19.7_27.3) | 26.4 (22.1_30.7) | 26.9 (22.5_31.5) | 32.5 (27.4_37.6) | 23.6 (19.7_27.6) | 22.2 (18.5_26) | 19.8 (16.4_23.3) | Lorestan |
| 23.2 (19.6_26.9) | 23.6 (19.8_27.4) | 23.7 (19.9_27.5) | 24 (20.2_27.8) | 23.6 (19.8_27.4) | 24.4 (20.5_28.4) | 25.8 (21.6_30) | 26.2 (21.9_30.6) | 31.5 (26.5_36.6) | 22.9 (19_26.8) | 21.4 (17.8_25.1) | 21.9 (18.2_25.8) | Gilan |
| 23.1 (19.6_26.8) | 23.5 (19.8_27.2) | 23.6 (19.9_27.3) | 23.9 (20.1_27.7) | 23.5 (19.8_27.3) | 24 (20.1_27.9) | 25.7 (21.5_29.9) | 26.1 (21.8_30.5) | 30.1 (25.3_35) | 22.7 (18.9_26.6) | 21.2 (17.6_24.9) | 20.7 (17.1_24.3) | QOM |
| 23.4 (19.7_27) | 23.7 (20_27.5) | 23.8 (20.1_27.6) | 24.2 (20.4_28) | 23.8 (20_27.6) | 25.6 (21.5_29.7) | 26 (21.8_30.3) | 26.5 (22.1_31) | 28.5 (23.9_33.1) | 23.1 (19.2_27) | 21.7 (18_25.4) | 20.8 (17.3_24.5) | Mazandaran |
| 24.1 (20.4_27.8) | 24.5 (20.7_28.4) | 24.7 (20.8_28.6) | 25.1 (21.2_29.1) | 24.8 (20.9_28.7) | 23 (19.3_26.8) | 27.3 (22.9_31.7) | 27.9 (23.3_32.5) | 30.4 (25.5_35.3) | 24.6 (20.5_28.7) | 23.2 (19.3_27.2) | 18.4 (15.2_21.7) | Zanjan |
| 23.5 (19.9_27.2) | 24.2 (20.4_28) | 24.6 (20.7_28.5) | 25.3 (21.4_29.3) | 25.3 (21.3_29.4) | 22.6 (18.9_26.3) | 28.8 (24.2_33.4) | 30 (25.2_35) | 30.7 (25.8_35.7) | 27.8 (23.3_32.4) | 27 (22.6_31.5) | 23.2 (19.2_27.2) | Markazi |
| 22.8 (19.2_26.4) | 23.4 (19.7_27.1) | 23.7 (20_27.5) | 24.4 (20.5_28.3) | 24.4 (20.5_28.3) | 25.3 (21.3_29.4) | 27.6 (23.2_32.1) | 28.7 (24.1_33.5) | 33 (27.8_38.2) | 26.4 (22.1_30.8) | 25.6 (21.3_29.9) | 23.7 (19.7_27.8) | Fars |
| 22.1 (18.7_25.6) | 22.7 (19.1_26.3) | 23 (19.4_26.7) | 23.6 (19.9_27.4) | 23.5 (19.8_27.3) | 22 (18.4_25.6) | 26.5 (22.2_30.9) | 27.5 (23_32.1) | 32.1 (27_37.2) | 25.2 (21.1_29.5) | 24.4 (20.3_28.5) | 21.7 (18_25.5) | Qazvin |
| 23.4 (19.8_27.1) | 24 (20.3_27.9) | 24.5 (20.6_28.3) | 25.2 (21.2_29.2) | 25.2 (21.2_29.2) | 21.9 (18.3_25.5) | 28.6 (24.1_33.3) | 29.8 (25_34.7) | 22.7 (18.9_26.5) | 27.6 (23.1_32.1) | 26.8 (22.4_31.3) | 22.6 (18.8_26.6) | Semnan |
| 25.1 (21.3_28.9) | 25.9 (21.9_29.9) | 26.4 (22.3_30.5) | 27.3 (23.1_31.5) | 27.4 (23.1_31.6) | 26.7 (22.5_30.9) | 31.3 (26.4_36.3) | 32.8 (27.6_38.1) | 26.7 (22.3_31.1) | 30.7 (25.8_35.6) | 30 (25.2_34.9) | 29.4 (24.6_34.3) | Sistan and Baluchistan |
| 21.9 (18.4_25.4) | 22.4 (18.8_26) | 22.7 (19.1_26.4) | 23.3 (19.6_27.1) | 23.2 (19.5_27) | 18.5 (15.4_21.7) | 26.1 (21.9_30.4) | 27.1 (22.7_31.6) | 25.9 (21.7_30.2) | 24.8 (20.7_28.9) | 23.9 (19.9_28) | 18.3 (15.1_21.5) | Khorasan, South |
| 21.9 (18.5_25.4) | 22.4 (18.8_26) | 22.7 (19.1_26.4) | 23.3 (19.6_27.1) | 23.2 (19.5_27) | 25.6 (21.5_29.7) | 26.2 (21.9_30.4) | 27.1 (22.7_31.6) | 30.1 (25.3_35) | 24.8 (20.7_28.9) | 23.9 (19.9_28) | 29.3 (24.5_34.1) | Ardabil |
| 16.8 (14_19.6) | 17.7 (14.7_20.7) | 18.5 (15.4_21.6) | 19.6 (16.4_22.8) | 20.2 (16.9_23.5) | 21.2 (17.7_24.7) | 24.4 (20.4_28.5) | 26.4 (22_30.8) | 33.5 (28.2_38.8) | 26.3 (22_30.7) | 26.6 (22.3_31.1) | 24.4 (20.3_28.5) | Isfahan |
| 14.2 (11.8_16.6) | 15 (12.4_17.6) | 15.6 (13_18.4) | 16.6 (13.8_19.4) | 17.1 (14.2_20) | 18 (14.9_21) | 20.7 (17.2_24.2) | 22.4 (18.6_26.2) | 25.2 (21.1_29.5) | 22.3 (18.5_26.1) | 22.5 (18.8_26.4) | 23.1 (19.2_27.1) | Kerman |
| 18.2 (15.3_21.3) | 19.2 (16.1_22.5) | 20.1 (16.8_23.4) | 21.3 (17.9_24.8) | 22 (18.4_25.6) | 23.8 (20_27.7) | 26.6 (22.3_31) | 28.7 (24.1_33.5) | 35.1 (29.7_40.6) | 28.6 (24_33.3) | 29 (24.3_33.7) | 30.6 (25.7_35.7) | Tehran |
| 17 (14.2_19.9) | 18 (15_21) | 18.8 (15.7_21.9) | 19.9 (16.6_23.2) | 20.5 (17.2_23.9) | 24.3 (20.4_28.3) | 24.9 (20.8_29) | 26.8 (22.4_31.3) | 31.6 (26.6_36.7) | 26.8 (22.4_31.2) | 27.1 (22.6_31.6) | 27.6 (23_32.2) | Azerbaijan, East |
| 16.2 (13.5_19) | 17.1 (14.2_20.1) | 17.9 (14.9_20.9) | 18.9 (15.8_22.1) | 19.5 (16.3_22.8) | 18.9 (15.8_22.1) | 23.7 (19.8_27.6) | 25.6 (21.3_29.9) | 30.2 (25.4_35.1) | 25.5 (21.3_29.8) | 25.8 (21.5_30.1) | 23.9 (19.9_28.1) | Khorasan, Razavi |
| 18.1 (15.2_21.1) | 19.1 (16_22.3) | 20 (16.7_23.3) | 21.2 (17.7_24.7) | 21.8 (18.3_25.4) | 26.1 (22_30.3) | 26.4 (22.2_30.8) | 28.6 (23.9_33.3) | 26.5 (22.2_30.9) | 28.5 (23.9_33.2) | 28.8 (24.2_33.6) | 34 (28.6_39.4) | Chahar Mahaal and Bakhtiari |
| 10.5 (8.7_12.4) | 12.4 (10.3_14.7) | 14.4 (11.9_16.9) | 16.7 (13.9_19.6) | 18.6 (15.5_21.8) | 20.7 (17.2_24.1) | 25.9 (21.7_30.1) | 29.6 (24.8_34.5) | 29.9 (25.1_34.7) | 32.8 (27.6_38) | 34.8 (29.3_40.3) | 37 (31.2_42.8) | Kohgiluyeh and Boyer-Ahmad |
| 10.5 (8.7_12.4) | 12.4 (10.3_14.7) | 14.4 (11.9_16.9) | 16.7 (13.9_19.6) | 18.6 (15.5_21.8) | 24.7 (20.7_28.7) | 25.9 (21.7_30.1) | 29.6 (24.8_34.5) | 29.2 (24.5_34) | 32.8 (27.6_38) | 34.8 (29.3_40.3) | 38.3 (32.3_44.3) | Azerbaijan, West |
| 10.5 (8.7_12.4) | 12.4 (10.3_14.7) | 14.4 (11.9_16.9) | 16.7 (13.9_19.6) | 18.6 (15.5_21.8) | 24.1 (20.2_28) | 25.9 (21.7_30.1) | 29.6 (24.8_34.5) | 33.9 (28.6_39.2) | 32.8 (27.6_38) | 34.8 (29.4_40.2) | 37 (31.2_42.8) | Alborz |
| 10.2 (8.4_12) | 12.3 (10.1_14.5) | 14.4 (11.9_16.9) | 16.8 (14_19.7) | 18.9 (15.8_22.1) | 19.1 (15.9_22.4) | 26.6 (22.3_31) | 30.6 (25.7_35.6) | 32.4 (27.3_37.6) | 34.1 (28.8_39.5) | 36.3 (30.6_41.9) | 40 (33.9_46.2) | Bushehr |
| 10.2 (8.4_12) | 12.3 (10.1_14.5) | 14.4 (11.9_16.9) | 16.8 (14_19.7) | 18.9 (15.8_22.1) | 24.6 (20.7_28.6) | 26.6 (22.3_31) | 30.6 (25.7_35.6) | 27.7 (23.2_32.3) | 34.1 (28.8_39.5) | 36.2 (30.6_41.9) | 40.1 (34_46.3) | Kurdistan |
| 10.2 (8.4_12) | 12.3 (10.1_14.5) | 14.4 (11.9_16.9) | 16.8 (14_19.7) | 18.9 (15.8_22.1) | 22.4 (18.7_26.1) | 26.6 (22.3_31) | 30.6 (25.7_35.6) | 28.2 (23.7_32.9) | 34.1 (28.8_39.5) | 36.2 (30.7_41.9) | 37.3 (31.5_43.2) | Hormozgan |

**TableS11.** Burden of Otitis media (DALY/100000) related to SHS exposure in Iran adolescents at the provincial level (2009-2020)

| **2020** | **2019** | **2018** | **2017** | **2016** | **2014** | **2013** | **2012** | **2011** | **2010** | **2010** | **2009** | **Province** |
| --- | --- | --- | --- | --- | --- | --- | --- | --- | --- | --- | --- | --- |
| 3.9 (2.4_5.6) | 3.8 (2.3_5.6) | 3.8 (2.3_5.5) | 3.7 (2.3_5.4) | 3.6 (2.2_5.3) | 3.6 (2.2_5.2) | 3.5 (2.2_5.2) | 3.5 (2.1_5.1) | 3.7 (2.3_5.4) | 3.3 (2_4.9) | 3.3 (2_4.8) | 3.1 (1.9_4.6) | National |
| 4.3 (2.6_6.2) | 4.1 (2.5_6) | 3.9 (2.4_5.7) | 3.7 (2.3_5.4) | 3.5 (2.1_5.1) | 3.9 (2.4_5.7) | 3.1 (1.9_4.5) | 2.9 (1.7_4.2) | 2.5 (1.5_3.7) | 2.4 (1.5_3.6) | 2.2 (1.3_3.2) | 1.6 (1_2.4) | Golestan |
| 4.2 (2.6_6.1) | 4 (2.5_5.9) | 3.8 (2.4_5.6) | 3.6 (2.2_5.3) | 3.4 (2.1_5) | 3.8 (2.3_5.5) | 3 (1.8_4.4) | 2.8 (1.7_4.1) | 2 (1.2_3) | 2.3 (1.4_3.4) | 2.1 (1.3_3.1) | 1.4 (0.8_2) | Ilam |
| 4.6 (2.9_6.8) | 4.5 (2.8_6.5) | 4.3 (2.6_6.3) | 4.1 (2.5_6) | 3.9 (2.4_5.7) | 4.1 (2.5_6) | 3.5 (2.2_5.1) | 3.3 (2_4.8) | 3.2 (2_4.7) | 2.9 (1.7_4.2) | 2.6 (1.6_3.9) | 1.9 (1.2_2.9) | Kermanshah |
| 4.7 (2.9_6.8) | 4.5 (2.8_6.6) | 4.3 (2.7_6.3) | 4.1 (2.6_6) | 4 (2.4_5.8) | 3.6 (2.2_5.2) | 3.6 (2.2_5.2) | 3.3 (2_4.9) | 2.6 (1.6_3.9) | 2.9 (1.8_4.3) | 2.7 (1.6_4) | 1.7 (1_2.6) | Khorasan, North |
| 4.8 (3_7) | 4.7 (2.9_6.8) | 4.5 (2.8_6.5) | 4.3 (2.7_6.3) | 4.1 (2.5_6) | 3.7 (2.3_5.4) | 3.7 (2.3_5.5) | 3.5 (2.2_5.1) | 4.3 (2.7_6.3) | 3.1 (1.9_4.5) | 2.9 (1.7_4.2) | 2.2 (1.3_3.3) | Hamadan |
| 4.8 (3_6.9) | 4.6 (2.8_6.7) | 4.4 (2.7_6.4) | 4.2 (2.6_6.2) | 4 (2.5_5.9) | 4 (2.4_5.8) | 3.6 (2.2_5.3) | 3.4 (2.1_5) | 4.6 (2.9_6.7) | 3 (1.8_4.4) | 2.8 (1.7_4.1) | 2.5 (1.5_3.8) | Yazd |
| 5 (3.1_7.2) | 4.8 (3_7) | 4.6 (2.9_6.7) | 4.4 (2.7_6.4) | 4.2 (2.6_6.2) | 4 (2.4_5.8) | 3.9 (2.4_5.7) | 3.6 (2.2_5.3) | 3.3 (2_4.8) | 3.2 (2_4.8) | 3 (1.8_4.5) | 2.8 (1.7_4.1) | Khuzestan |
| 4.7 (2.9_6.8) | 4.5 (2.8_6.6) | 4.3 (2.7_6.3) | 4.1 (2.6_6.1) | 4 (2.4_5.8) | 3.6 (2.2_5.2) | 3.6 (2.2_5.2) | 3.3 (2.1_4.9) | 4.1 (2.6_6) | 2.9 (1.8_4.3) | 2.7 (1.6_4) | 2.4 (1.4_3.5) | Lorestan |
| 4.6 (2.9_6.7) | 4.4 (2.7_6.5) | 4.3 (2.6_6.2) | 4.1 (2.5_5.9) | 3.9 (2.4_5.7) | 3.7 (2.3_5.4) | 3.5 (2.1_5.1) | 3.2 (2_4.8) | 4 (2.5_5.8) | 2.8 (1.7_4.2) | 2.6 (1.6_3.8) | 2.6 (1.6_3.9) | Gilan |
| 4.6 (2.9_6.7) | 4.4 (2.7_6.4) | 4.2 (2.6_6.2) | 4 (2.5_5.9) | 3.9 (2.4_5.6) | 3.6 (2.2_5.3) | 3.5 (2.1_5.1) | 3.2 (2_4.7) | 3.8 (2.3_5.6) | 2.8 (1.7_4.1) | 2.6 (1.6_3.8) | 2.5 (1.5_3.7) | QOM |
| 4.6 (2.9_6.8) | 4.5 (2.8_6.5) | 4.3 (2.6_6.3) | 4.1 (2.5_6) | 3.9 (2.4_5.7) | 3.9 (2.4_5.7) | 3.5 (2.2_5.2) | 3.3 (2_4.8) | 3.6 (2.2_5.2) | 2.9 (1.7_4.2) | 2.6 (1.6_3.9) | 2.5 (1.5_3.7) | Mazandaran |
| 4.8 (3_7) | 4.6 (2.9_6.7) | 4.5 (2.8_6.5) | 4.3 (2.6_6.2) | 4.1 (2.5_6) | 3.5 (2.1_5.1) | 3.7 (2.3_5.4) | 3.5 (2.1_5.1) | 3.8 (2.4_5.6) | 3.1 (1.9_4.5) | 2.8 (1.7_4.2) | 2.2 (1.3_3.2) | Zanjan |
| 4.7 (2.9_6.8) | 4.6 (2.8_6.6) | 4.4 (2.7_6.5) | 4.3 (2.7_6.3) | 4.2 (2.6_6.1) | 3.4 (2.1_5) | 3.9 (2.4_5.7) | 3.8 (2.3_5.5) | 3.9 (2.4_5.7) | 3.5 (2.1_5.1) | 3.4 (2.1_4.9) | 2.8 (1.7_4.1) | Markazi |
| 4.5 (2.8_6.6) | 4.4 (2.7_6.4) | 4.3 (2.6_6.2) | 4.1 (2.6_6) | 4 (2.5_5.9) | 3.9 (2.4_5.7) | 3.7 (2.3_5.5) | 3.6 (2.2_5.3) | 4.2 (2.6_6.1) | 3.3 (2_4.8) | 3.2 (1.9_4.6) | 2.9 (1.7_4.2) | Fars |
| 4.4 (2.7_6.4) | 4.2 (2.6_6.2) | 4.1 (2.5_6) | 4 (2.5_5.8) | 3.9 (2.4_5.6) | 3.3 (2_4.9) | 3.6 (2.2_5.2) | 3.4 (2.1_5) | 4.1 (2.5_6) | 3.1 (1.9_4.6) | 3 (1.8_4.4) | 2.6 (1.6_3.9) | Qazvin |
| 4.7 (2.9_6.8) | 4.5 (2.8_6.6) | 4.4 (2.7_6.4) | 4.3 (2.6_6.2) | 4.2 (2.6_6.1) | 3.3 (2_4.8) | 3.9 (2.4_5.7) | 3.7 (2.3_5.5) | 2.8 (1.7_4.1) | 3.5 (2.1_5.1) | 3.3 (2_4.9) | 2.7 (1.7_4) | Semnan |
| 5 (3.1_7.3) | 4.9 (3.1_7.1) | 4.8 (3_7) | 4.7 (2.9_6.8) | 4.6 (2.8_6.7) | 4.1 (2.5_6) | 4.3 (2.7_6.3) | 4.2 (2.6_6.1) | 3.3 (2_4.9) | 3.9 (2.4_5.7) | 3.8 (2.3_5.5) | 3.6 (2.2_5.3) | Sistan and Baluchistan |
| 4.3 (2.7_6.3) | 4.2 (2.6_6.1) | 4.1 (2.5_5.9) | 3.9 (2.4_5.7) | 3.8 (2.3_5.6) | 2.8 (1.7_4.1) | 3.5 (2.2_5.2) | 3.4 (2.1_4.9) | 3.2 (2_4.7) | 3.1 (1.9_4.5) | 2.9 (1.8_4.3) | 2.2 (1.3_3.2) | Khorasan, South |
| 4.3 (2.7_6.3) | 4.2 (2.6_6.1) | 4.1 (2.5_5.9) | 3.9 (2.4_5.7) | 3.8 (2.3_5.6) | 3.9 (2.4_5.7) | 3.5 (2.2_5.2) | 3.4 (2.1_4.9) | 3.8 (2.3_5.6) | 3.1 (1.9_4.5) | 2.9 (1.8_4.3) | 3.6 (2.2_5.3) | Ardabil |
| 3.2 (2_4.7) | 3.2 (2_4.7) | 3.2 (2_4.8) | 3.2 (2_4.8) | 3.3 (2_4.8) | 3.2 (1.9_4.7) | 3.3 (2_4.8) | 3.3 (2_4.8) | 4.3 (2.6_6.2) | 3.3 (2_4.8) | 3.3 (2_4.9) | 3 (1.8_4.4) | Isfahan |
| 2.7 (1.6_4) | 2.7 (1.6_4) | 2.7 (1.6_4) | 2.7 (1.7_4) | 2.7 (1.7_4) | 2.7 (1.6_3.9) | 2.7 (1.7_4) | 2.7 (1.7_4) | 3.1 (1.9_4.6) | 2.7 (1.7_4) | 2.8 (1.7_4.1) | 2.8 (1.7_4.1) | Kerman |
| 3.5 (2.2_5.2) | 3.5 (2.2_5.2) | 3.6 (2.2_5.2) | 3.6 (2.2_5.2) | 3.6 (2.2_5.2) | 3.6 (2.2_5.3) | 3.6 (2.2_5.3) | 3.6 (2.2_5.2) | 4.5 (2.8_6.6) | 3.6 (2.2_5.3) | 3.6 (2.2_5.3) | 3.8 (2.3_5.6) | Tehran |
| 3.3 (2_4.8) | 3.3 (2_4.8) | 3.3 (2_4.8) | 3.3 (2_4.8) | 3.3 (2_4.9) | 3.7 (2.3_5.4) | 3.3 (2_4.9) | 3.3 (2_4.9) | 4 (2.5_5.9) | 3.4 (2.1_4.9) | 3.4 (2.1_4.9) | 3.4 (2.1_5) | Azerbaijan, East |
| 3.1 (1.9_4.6) | 3.1 (1.9_4.6) | 3.1 (1.9_4.6) | 3.1 (1.9_4.6) | 3.1 (1.9_4.6) | 2.8 (1.7_4.2) | 3.2 (1.9_4.7) | 3.2 (1.9_4.6) | 3.8 (2.4_5.6) | 3.2 (1.9_4.7) | 3.2 (1.9_4.7) | 2.9 (1.8_4.3) | Khorasan, Razavi |
| 3.5 (2.2_5.1) | 3.5 (2.2_5.1) | 3.5 (2.2_5.2) | 3.5 (2.2_5.2) | 3.6 (2.2_5.2) | 4 (2.5_5.9) | 3.6 (2.2_5.2) | 3.6 (2.2_5.2) | 3.3 (2_4.8) | 3.6 (2.2_5.3) | 3.6 (2.2_5.3) | 4.3 (2.6_6.2) | Chahar Mahaal and Bakhtiari |
| 1.9 (1.2_2.9) | 2.2 (1.3_3.3) | 2.5 (1.5_3.6) | 2.7 (1.7_4) | 3 (1.8_4.4) | 3.1 (1.9_4.6) | 3.5 (2.1_5.1) | 3.7 (2.3_5.4) | 3.8 (2.3_5.5) | 4.2 (2.6_6.1) | 4.4 (2.7_6.5) | 4.7 (2.9_6.8) | Kohgiluyeh and Boyer-Ahmad |
| 1.9 (1.2_2.9) | 2.2 (1.3_3.3) | 2.5 (1.5_3.6) | 2.7 (1.7_4) | 3 (1.8_4.4) | 3.8 (2.3_5.5) | 3.5 (2.1_5.1) | 3.7 (2.3_5.4) | 3.7 (2.3_5.4) | 4.2 (2.6_6.1) | 4.4 (2.7_6.5) | 4.9 (3_7.1) | Azerbaijan, West |
| 1.9 (1.2_2.9) | 2.2 (1.3_3.3) | 2.5 (1.5_3.6) | 2.7 (1.7_4) | 3 (1.8_4.4) | 3.7 (2.2_5.4) | 3.5 (2.1_5.1) | 3.7 (2.3_5.4) | 4.3 (2.7_6.3) | 4.2 (2.6_6.1) | 4.4 (2.7_6.5) | 4.7 (2.9_6.8) | Alborz |
| 1.9 (1.1_2.8) | 2.2 (1.3_3.2) | 2.5 (1.5_3.6) | 2.8 (1.7_4.1) | 3 (1.9_4.5) | 2.9 (1.7_4.2) | 3.6 (2.2_5.3) | 3.8 (2.4_5.6) | 4.1 (2.5_6) | 4.4 (2.7_6.4) | 4.6 (2.9_6.8) | 5.1 (3.2_7.4) | Bushehr |
| 1.9 (1.1_2.8) | 2.2 (1.3_3.2) | 2.5 (1.5_3.6) | 2.8 (1.7_4.1) | 3 (1.9_4.5) | 3.8 (2.3_5.5) | 3.6 (2.2_5.3) | 3.8 (2.4_5.6) | 3.5 (2.1_5.1) | 4.4 (2.7_6.4) | 4.6 (2.9_6.8) | 5.1 (3.2_7.5) | Kurdistan |
| 1.9 (1.1_2.8) | 2.2 (1.3_3.2) | 2.5 (1.5_3.6) | 2.8 (1.7_4.1) | 3 (1.9_4.5) | 3.4 (2.1_5) | 3.6 (2.2_5.3) | 3.8 (2.4_5.6) | 3.5 (2.2_5.2) | 4.4 (2.7_6.4) | 4.6 (2.9_6.8) | 4.7 (2.9_6.9) | Hormozgan |
